# Clinical Features and Prognosis in ANCA Associated Vasculitis Patients with Acute Kidney Injury

**DOI:** 10.34172/aim.2022.39

**Published:** 2022-04-01

**Authors:** Yue Chen, Yuqiu Lu, Ying Zhou, Chen Yu

**Affiliations:** ^1^Department of Nephrology, Tongji Hospital, Tongji University School of Medicine, Shanghai, China

**Keywords:** Acute kidney injury, Anti-neutrophil cytoplasmic antibody associated va, Prognosis, Risk factors

## Abstract

**Background:**

Renal involvement in patients with anti-neutrophil cytoplasmic antibody (ANCA) associated vasculitis (AAV) often presents as acute kidney injury (AKI), which is closely related to the prognosis of critically ill patients. Nevertheless, there are few researches on the subgroup of AVV patients with AKI. The study aimed to explore clinical features and prognosis in AAV patients with AKI.

**Methods:**

A retrospective analysis of AAV patients was conducted. The enrolled patients were grouped according to whether AKI on admission occurred or not. Demographic, clinical data and follow-up records were gathered from medical histories. Survival rates of AAV patients with AKI and risk factors of all AAV patients were studied.

**Results:**

(1) In the AKI group, the levels of hemoglobin, evaluated glomerular filtration rate (eGFR), serum albumin and complement 3 were significantly lower (*P*<0.05); the proportions of microscopic polyangiitis (MPA) and levels of serum creatinine (SCr) on admission, red blood cell (RBC) counts in urine, 24-hour urine protein excretion (UPE) and Birmingham Vasculitis Activity Score (BVAS) were significantly higher (*P*<0.05). There was a significantly lower incidence of otorhinolaryngologic involvement in the AKI group (*P*<0.05). (2) There were significantly lower survival rates and renal survival rates in the AKI group (*P*<0.05). (3) Higher creatinine and AKI were risk factors for poor prognosis in AAV patients.

**Conclusion:**

AAV patients with AKI have more severe kidney damage, higher disease activity and worse prognosis. More attention should be paid to AAV patients with AKI for both remission induction and infection prevention.

## Introduction

 Anti-neutrophil cytoplasmic antibody (ANCA) associated vasculitis (AAV) is a rare autoimmune disease. ANCA positivity and inflammation of small vessels are its main characteristics. AAV includes three different entities: microscopic polyangiitis (MPA), granulomatosis with polyangiitis (GPA), and eosinophilic GPA (eGPA).^1,2^ AAV could affect any system of the body, and 25%–75% of AAV patients have renal involvement.^3^ In spite of a general improvement in remission and survival of AAV, the renal involvement in AAV patients suggests higher mortality and morbidity rates.^4^ Renal involvement in AAV patients often manifests as acute kidney injury (AKI), which is mostly caused by glomerular vascular damage.^5,6^ Notably, the occurrence of AKI is closely related to the prognosis and mortality in critically illpatients.^7^ Therefore, more attention should be paid to AAV patients with AKI.

 However, to the best of our knowledge, there are few researches on the subgroup of AVV patients with AKI. This study explored clinical features and prognosis in AAV patients with AKI retrospectively. It can provide a good grasp of AAV patients with AKI and improve the outcomes.

## Materials and Methods

###  Subjects

 A retrospective observational research was done in our center, Tongji Hospital affiliated to Tongji University. During the period from June 1, 2011 to June 1, 2019, adult patients with newly diagnosed AAV were enrolled. All patients met the diagnostic criteria of AAV, which have been proposed at the Chapel Hill meeting.^8^ The exclusion criteria were: patients with secondary vasculitis, patients complicated by other autoimmune diseases, patients who were followed up for less than 3 months and failed to reach the endpoints.

 The study conformed to the Helsinki Declaration. Oral informed consents for this study were obtained from the patients or their guardians.

###  Measurements

 The medical records were reviewed at the first diagnosis of AAV, including age, sex, clinical features, treatments, ANCA serology, serum creatinine (SCr) on admission, 24-hour urine protein excretion (UPE), red blood cell (RBC) counts in urine, serum albumin (Salb), hemoglobin (HB), C-reactive protein (CRP), peripheral white blood cell (WBC) counts, peripheral platelet counts, and complement 3 (C3). The Modification of Diet in Renal Disease (MDRD) formula was used to calculate the evaluated glomerular filtration rate (eGFR).^9^ The activity of AAV was evaluated by the Birmingham Vasculitis Activity Score (BVAS).^10^

###  Treatment Schemes

 The initial immunosuppressive therapy included steroids alone (prednisone), and steroids plus immunosuppressants (such as cyclophosphamide, methotrexate and leflunomide). The initial oral dose of prednisone was 0.6–1 mg/(kg/d). The dose of prednisone was gradually reduced after 4–6 weeks, and then it decreased to 10 mg/d after 12 weeks. Some patients were given methylprednisolone (0.5–1.0 g/d) intravenously for 3 days, and then prednisone orally. The dose of cyclophosphamide (CTX) was 0.6–0.8 g/m^2^. Following administration of CTX for 6–8 months, mycophenolate mofetil or thiazolpurine were used to maintain clinical remission. According to the guidelines (11), plasma exchange was administered to patients with severe AKI requiring dialysis at onset, patients with diffuse alveolar hemorrhage, and patients diagnosed with anti-glomerular basement membrane (GBM) antibody disease.

###  Groups 

 The definition of AKI was: SCr increased by 26.5 μmol/L in 48 hours; or urine output less than 0.5 mL (kg/h) for 6 hours; or SCr increased by 50% higher than baseline, with the increase occurring in 7 days.^11^ AKI was staged based on the severity of SCr on admission according to KDIGO guidelines.^11^ In this study, the lowest value of SCr in the past 3 months or in 3 months after admission was selected as the baseline value of SCr, which could not be identified in 7 days before admission. The enrolled patients were grouped according to whether AKI on admission occurred or not, forming the AKI group and non- AKI group, respectively.

###  Clinical Outcomes

 The endpoint was defined as all-cause death or end-stage renal disease (ESRD). ESRD was defined as maintenance dialysis (duration of dialysis ≥ 3months). The follow-up started from the definite diagnosis of AAV. The end time of follow-up was October 31, 2019 or the endpoint happening. The follow-up data included survival status, ANCA level, SCr, UPE, Salb and need for dialysis.

###  Statistical Methods

 The SPSS statistical software (v22.0, Chicago, IL, USA) was used to perform all statistical analyses. Normally distributed numerical variables were expressed in mean ± standard deviation (SD) and analyzed by the independent *t *test; non-normally distributed data was expressed in median with interquartile range (IQR) and analyzed by the non-parametric Mann-Whitney U test. Normality assumption was assessed by the Shapiro-Wilk test. Before the independent t test, homogeneity of variance was tested. Categorical variables were expressed in numbers (percentages) and analyzed by the chi-square test or Fisher’s exact probability. Kaplan-Meier methods were used to calculate the survival rates, the difference of which was compared by the log-rank test. We tested theproportional hazards hypothesis with the Schoenfeld partial residual method for continuous variables and with the log minus log method for categorical variables. After the proportional hazard assumption was confirmed, the risk factors were determined using COX regression analysis. Firstly, univariate COX analysis was conducted to identify variables. Subsequently, variables with *P* value < 0.10 were selected and entered into multivariate analysis. Hazard ratio (HR) and 95% confidence intervals (CIs) were calculated for endpoint events. If the predictive index was a numerical variable, we plotted the receiver operating characteristic (ROC) curve and calculated the area under the ROC curve (AUC). The point closest to the upper left corner of the ROC curve was the cut-off value. For *P* values less than 0.05, the difference was considered to be significant statistically.

## Results

###  Basic Information

 In total, 85 patients were newly diagnosed with AAV. Among them, one case was secondary to rheumatoid arthritis, and 13 patients were followed up for less than three months and failed to reach the endpoint. The 71 patients enrolled in this study consisted of 38 males and 33 females. The age ranged from 33 to 92 years. The average age was 69.8. And 60 patients (84.5%) were elderly (≥ 60 years). ANCA serology was positive in all patients; 65 patients (91.5%) were myeloperoxidase (MPO)-ANCA single-positive, 5 (7.0%) were proteinase 3 (PR3)-ANCA single-positive, and 1 (1.4%) was MPO and PR3-ANCA double-positive. According to the vasculitis disease classification, 64 patients (90.1%) were diagnosed as MPA, whereas 7 (9.9%) as GPA. Forty patients (56.3%) suffered from AKI on admission. The numbers of patients with AKI stage 1–3 was 15, 7 and 18, respectively. Twenty-five (80.6%) of 31 patients without AKI had proteinuria, hematuria or renal insufficiency.

###  Comparison of Baseline Characteristics and Prognosis in Two Groups

####  Baseline Clinical Characteristics

 Compared with the non-AKI group, the levels of HB, eGFR, Salb and C3 in the AKI group were significantly lower (*P*< 0.05), while the proportions of MPA, SCr on admission, RBC counts in urine, UPE and BVAS in the AKI group were significantly higher (*P*< 0.05). Between the groups, there were no differences in age, sex, the proportion of the elderly, peripheral WBC counts, neutrophil ratio, CRP and the proportion of increased RF (*P*> 0.05) (Table 1).

**Table 1:** Clinical Features of AAV Patients in Two Groups

**Variables**	**Total**	**AKI Group**	**Non-AKI Group**	***P*** **Value** ^a^
Patient number, *n* (%)	71 (100)	40 (56.3)	31 (43.7)	
Age (years)	69.8 ± 1.3	70.5 ± 9.0	68.9 ± 13.6	0.554
Age ≥ 60 years, *n* (%)	60 (84.5)	36 (90.0)	24 (77.4)	0.146
Male, *n* (%)	38 (53.5)	23 (57.5)	15 (48.4)	0.445
Clinical classifications				0.038
MPA, *n* (%)	64 (90.1)	39(97.5)	25 (80.6)	
GPA, *n* (%)	7 (9.9)	1 (2.5)	6 (19.4)	
Anti-GBM positive, *n* (%)	2 (2.8)	2 (5)	0 (0)	
Median peripheral WBC counts (× 10^9^/L, IQR)	9.1 (6.5–12.7)	9.3 (6.5–11.5)	8.4 (6.8–13.9)	0.931
HB (g/L)	99.2 ± 2.4	91.1 ± 17.8	109.7 ± 18.2	< 0.001
Median neutrophil ratios (%, IQR)	76.9 (69.9–84.2)	77.5 (71.4–85.3)	75.9 (65.1–83.6)	0.151
Median serum CRP (mg/L, IQR)	53.5 (7.0–103.0)	55.8 (11.4–102.0)	53.5 (5.0–109.9)	0.558
Median SCr on admission (μmol/L, IQR)	147.0 (76.0–375.0)	292.0 (159.5–519.5)	75.0 (65.0–84.0)	< 0.001
Median eGFR on admission (mL/min·1.73 m^2^, IQR)	35.8(10.8–82.5)	17.5 (8.3–31.3)	86.0 (70.3–96.5)	< 0.001
Salb (g/L)	29.4 ± 0.7	27.9 ± 5.2	31.3 ± 5.9	0.013
Median RBC counts in urine (/μL, IQR)	58 (6–342)	249 (54–803)	5(0–30)	< 0.001
Median UPE (g, IQR)	0.9 (0.3–2.0)	1.6 (0.7–3.9)	0.3 (0.1–0.6)	< 0.001
BVAS	18.6 ± 0.7	20.4 ± 4.3	16.3 ± 6.4	0.002
C3 (g/L)	1.0 ± 0.0	1.0 ± 0.2	1.1 ± 0.3	0.047
Increased RF, *n* (%)	28 (39.4)	17 (42.5)	11 (35.5)	0.549
Renal involvement, *n* (%)	65 (91.5)	40 (100.0)	25 (80.6)	

AKI, acute kidney injury; GPA, granulomatosis with polyangiitis; MPA, microscopic polyangiitis; GBM, glomerular basement membrane; WBC, white blood cells; HB, hemoglobin; CRP, C-reactive protein; SCr, serum creatinine; eGFR, estimated glomerular filtration rate; Salb, serum albumin; RBC, red blood cell; UPE, 24h urine protein excretion; BVAS, Birmingham Vasculitis Activity Score; C3, Complement 3; RF, rheumatoid factor. ^a^ Comparison between the two groups: t-test for continuous variables (age, HB, Salb, BVAS and C3); Chi-square test or Fisher exact probability for variables ( age ≥ 60 years, male, clinical classifications, and increased RF); Mann-Whitney U test for variables (peripheral WBC counts, neutrophil ratios, serum CRP, SCr on admission, eGFR on admission, UPE, RBC counts in urine).

####  Extrarenal Involvement

 As shown in Table 2, AAV patients in the AKI group had a significantly lower incidence of otorhinolaryngologic involvement (*P*< 0.05). No significant intergroup differences were observed in terms of the incidence of non-specific manifestations, skin manifestations, respiratory system, cardiovascular system and nervous system involvement. In the AKI group, two patients had digestive involvement, including acute pancreatitis and abdominal pain of unknown causes.

**Table 2:** Extrarenal Involvement of AAV Patients in the Two Groups

**Extrarenal Involvement**	**AKI Group**	**Non-AKI Group**	***P *** **Value** ^a^
Non-specific manifestations, *n* (%)	28 (70.0)	27 (87.1)	0.087
Skin, *n* (%)	1 (2.5)	5 (16.1)	0.079
Otorhinolaryngologic, *n* (%)	4 (10.0)	9 (29.0)	0.040
Respiratory system, *n* (%)	39 (97.5)	29 (93.5)	0.577
Cardiovascular system, *n* (%)	7 (17.5)	3 (9.7)	0.551
Nervous system, *n* (%)	3 (7.5)	4 (12.9)	0.691
Digestive system, *n* (%)	2 (2.5)	0 (0)	

AKI, acute kidney injury. ^a^ Comparison between two groups by using chi-square test or Fisher exact probability.

####  Initial Treatment Schemes

 As shown in Table 3, there was a significantly higher proportion of treatment with steroids plus immunosuppressants in the AKI group. Seven patients in the AKI group received plasma exchange, compared to none in the non-AKI group. The proportion of treatment with steroids alone was similar between the two groups (Table 3).

**Table 3:** Treatment of AAV Patients in the Two Groups

**Treatment Schemes**	**AKI Group**	**Non-AKI Group **	***P *** **Value** ^a^
Steroids alone, *n* (%)	2(5.0)	7(22.6)	0.065
Steroids plus immunosuppressants, *n *(%)	36(90.0)	21(67.7)	0.019
Plasma exchange, *n* (%)	7 (17.5)	0 (0)	

AKI, acute kidney injury. ^a^ Comparison between the two groups by Fisher exact probability.

####  Prognosis

**Figure 1:**
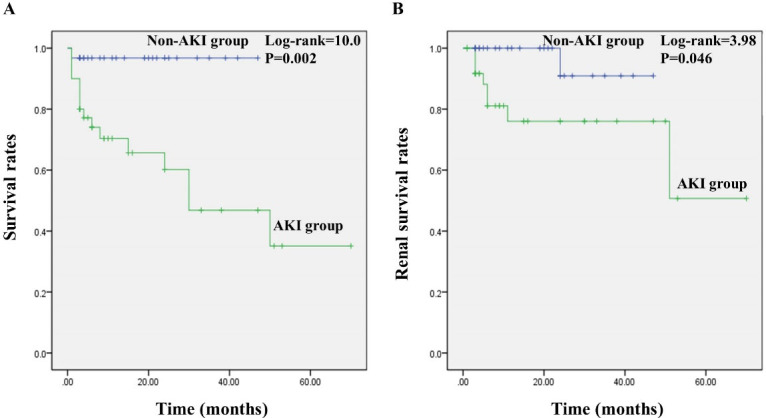
Survival Analyses of AAV Patients in Two Groups.** (A)** survival rates of patients; (**B)** renal survival rates. Kaplan-Meier method and the log-rank test were used to compare survival curves.

 The follow-up period was 1–70 months. Table 4 presents the endpoint events during the follow-up period. In the AKI group, the incidence of endpoints, death or ESRD was significantly higher (*P*< 0.05) (Table 4). The death causes in the AKI group included pulmonary infectious disease (11, 68.8%), cerebrovascular events (2, 12.5%), sudden cardiac death (2, 12.5%) and relapse of vasculitis (1, 6.2%). As shown in Figure 1, there were significantly lower survival rates of patients and renal survival rates in the AKI group (*P*< 0.05).

**Table 4:** Prognosis of AAV Patients in the Two Groups

	**AKI Group**	**Non-AKI Group**	***P *** **Value** ^a^
Median time of follow-up (months, IQR)	7 (3–24)	19 (4–24)	0.30
Endpoints, *n* (%)	24 (60.0)	2 (6.5)	< 0.001
Death, *n* (%)	16 (40.0)	1 (3.2)	< 0.001
End-stage renal disease, *n* (%)	8 (20.0)	1 (3.2)	0.035

AKI, acute kidney injury; IQR, interquartile range. ^a^ Fisher exact probability for variables (the incidences of endpoints, death or end-stage renal disease); Mann-Whitney U test for the variable (time of follow-up).

###  Cox Regression Analysis for Risk Factors of Endpoints in AAV Patients

**Figure 2:**
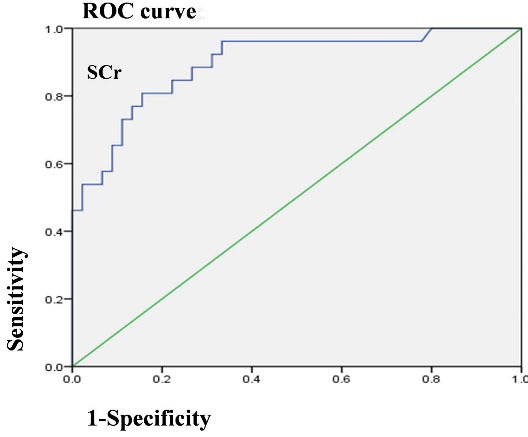
Receiver Operating Characteristic Curve for SCr on Admission in AAV Patients.

 The variables in the univariate COX analysis included age, sex, BVAS, with AKI, HB, peripheral WBC counts, neutrophil ratios, serum CRP, Salb, SCr on admission, C3, C4, RF, ESR, UPE and hematuria. Age, SCr on admission, with AKI, BVAS, HB, neutrophil ratios, C3 and hematuria were related to the endpoints. Finally, higher SCr on admission and AKI were determined as independent risk factors for endpoints in AAV patients (Table 5). Furthermore, the ROC curve was constructed for SCr on admission (Figure 2). The AUC of SCr on admission was 0.895 (95% CI: 0.817–0.973), and the threshold of SCr on admission was 192 μmol/L. Moreover, the sensitivity and specificity to predict endpoints were 80.8% (95% CI: 0.600–0.927) and 84.4% (95% CI: 0.699–0.930), respectively.

**Table 5:** Risk Factors for Endpoints in AAV Patients by COX Analysis

	**Univariate Analysis**			**Multivariable Analysis**		
	***P *** **Value**	**HR**	**95% CI**	***P *** **Value**	**HR**	**95% CI**
SCr on admission (μmol/L)	0.000	1.003	(1.002–1.004)	0.002	1.002	(1.001–1.004)
With AKI	0.003	9.319	(2.189–39.685)	0.045	5.879	(1.184–24.828)
Age (years)	0.026	1.048	(1.005–1.091)			
BVAS	0.026	1.081	(1.009–1.158)			
HB (g/L)	0.011	0.970	(0.948–0.993)			
Neutrophil ratios (%)	0.013	1.056	(1.012–1.103)			
C3 (g/L)	0.008	0.119	(0.025–0.577)			
Hematuria	0.064	3.924	(0.922–16.695)			

CI, confidence interval; HR, hazard ratio; SCr, Serum creatinine; AKI, acute kidney injury; BVAS, Birmingham Vasculitis Activity Score; HB, hemoglobin; C3, Complement 3. COX analysis was used to determine risk factors.

## Discussion

 This single-center study presented the comprehensive clinical information of AAV patients with AKI over an 8-year period in an economically developed city, which has the highest rate of aging in China. Moreover, the possible risk factors of poor prognosis were analyzed in the cohort of all AAV patients. This current study demonstrated that AAV patients with AKI had more severe kidney damage, higher disease activity and worse prognosis. Furthermore, this study suggested that higher SCr on admission and AKI were the risk factors for endpoints among AAV patients.

 AAV is one of common causes of secondary kidney diseases in the elderly. Our previous study on elderly patients undergoing renal biopsy found that AAV was the most common cause of secondary kidney diseases.^12^ AAV patients in this study had an average age of nearly 70, and 84.5% of them were elderly. Previous studies reported that GPA was most common in Europe, while MPA was dominant in Asia.^2^ The present study demonstrated that, among different subtypes of AAV, MPA accounted for 90.1%, which was close to the rate of 90.5% that was reported by another single-center study in China.^8^ AAV patients with renal involvement present with proteinuria, hematuria and renal dysfunction to varying degrees. Previous studies suggested that renal function at onset was related to the risk of ESRD and death in AAV patients.^13^ Furthermore, renal dysfunction in AAV patients is often manifested as AKI. Therefore, it has an important clinical significance in studying the subgroup of AAV patients with AKI.

 The current study demonstrated that AAV patients with AKI had lower levels of HB and Salb, which might be caused by the reduction of erythropoietin, insufficient intake of nutrients and inflammatory conditions. Notably, AAV patients with AKI had lower levels of C3, which was caused by large consumption of C3 due to complement activation. Moreover, clearance of immune complex is weakened after large consumption of C3, which aggravates the kidney injury.^3^ This study also found that AAV patients with AKI had more urine protein excretion, more severe hematuria and higher disease activity. This finding suggests that AAV patients with AKI are susceptible to more serious kidney damage and disease activity.

 The current study suggests that AAV patients with AKI have a lower incidence of otorhinolaryngologic involvement. Therefore, it may be suggested that AAV patients with otorhinolaryngologic involvement might have milder kidney injury. Recent evidence demonstrated that otorhinolaryngologic involvement is significantly related with lower levels of mortality in patients with GPA and eGPA.^14^ Also, “no otorhinolaryngologic involvement” is a scoring criterion in the Five Factor Score of the prognostic assessment scale for AAV patients, which was associated with poor prognosis of AAV patients.^15^

 Previous research showed that the leading cause of death in AAV patients was infections, among which pneumonia was the most common. In addition, severe infections often occurred within half a year of diagnosis.^16,17^ Likewise, our study found that 70.6% of deaths were due to pulmonary infections in AAV patients, and 75.0% of deaths from pulmonary infection occurred within half a year of diagnosis. Notably, this study demonstrated that more AAV patients with AKI need to be treated with steroids and immunosuppressants. Furthermore, the probability of infectious diseases increased in patients with AKI. Therefore, for AAV patients with AKI, it was necessary to weigh the pros and cons of immunosuppressive therapy. More attentions should be paid to individualized treatment and prevention of infectious diseases.

 It was confirmed that age and SCr on admission were closely related to the prognosis of AAV patients.^18^ Moreover, this study found that AKI was another risk factor for the endpoints in AAV patients. Furthermore, AAV patients with AKI had a lower survival rate and renal survival rate. Although the survival rate of AAV patients has been significantly improved with the application of immunosuppressive therapy, standard treatment is still important for prognosis of AAV patients.^6^ Unfortunately, the present study was limited by the number of patients and failed to conduct subgroup analysis of treatments. More research is needed to focus on the treatment of AAV and prevention of infections.

 There were several shortcomings in our study. First, this study was retrospective. Selection bias existed because there were no standardized treatments that might affect the curative effect and prognosis. Second, it had a small sample size. It was challenging to make timely diagnosis for various presenting symptoms of AAV. The correlation between AKI stage and endpoints could not be analyzed. Third, it had a small frequency of positive events, which might cause larger statistical error. Some patients might have reached the endpoints, but they were not included in this study due to loss to follow-up. Therefore, we need to expand the sample size and design a prospective trial for further research.

 In conclusion, AAV patients with AKI have more severe kidney damage, higher disease activity and worse prognosis. Therefore, for AAV patients with AKI, more attention should be paid to the induction of remission, and the prevention of infections is also very important.
